# Supplementary material for: Contamination of Fresh Produce with Antibiotic-Resistant Bacteria and Associated Risks to Human Health: A Scoping Review
Source: Int J Environ Res Public Health. 2021 Dec 30;19(1):360. doi: 10.3390/ijerph19010360 (PMC8744955; doi:10.3390/ijerph19010360)
Supplement: Supplementary file 1 [file ijerph-19-00360-s001.zip › ijerph-1424025-supplementary.pdf]

Supplementary Information:

Table S1: Preferred Reporting Items for Systematic reviews and Meta-Analyses extension for Scoping Reviews (PRISMA-ScR) Checklist

Section	Item	PRISMA-ScR Checklist Item	Reported on Page #
		TITLE	
Title	1	Identify the report as a scoping review.	1
		ABSTRACT	
Structured summary	2	Provide a structured summary that includes (as applicable): background, objectives, eligibility criteria, sources of evidence, charting methods, results, and conclusions that relate to the review questions and objectives.	1
		INTRODUCTION	
Rationale	3	Describe the rationale for the review in the context of what is already known. Explain why the review questions/objectives lend themselves to a scoping review approach.	2
Objectives	4	Provide an explicit statement of the questions and objectives being addressed with reference to their key elements (e.g., population or participants, concepts, and context) or other relevant key elements used to conceptualize the review questions and/or objectives.	2
		METHODS	
Protocol and registration	5	Indicate whether a review protocol exists; state if and where it can be accessed (e.g., a Web address); and if available, provide registration information, including the registration number.	A brief protocol has developed, but not registered Page 5, table 1
Eligibility criteria	6	Specify characteristics of the sources of evidence used as eligibility criteria (e.g., years considered, language, and publication status), and provide a rationale.	3-4 (table 1)
Information sources	7	Describe all information sources in the search (e.g., databases with dates of coverage and contact with authors to identify additional sources), as well as the date the most recent search was executed.	2 & 3
Search	8	Present the full electronic search strategy for at least 1 database, including any limits used, such that it could be repeated.	18 (table S2)
Selection of sources of evidence	9	State the process for selecting sources of evidence (i.e., screening and eligibility) included in the scoping review.	3 to 5
Data charting process	10	Describe the methods of charting data from the included sources of evidence (e.g., calibrated forms or forms that have been tested by the team before their use, and whether data charting was done independently or in duplicate) and any processes for obtaining and confirming data from investigators.	4 to 5
Data items	11	List and define all variables for which data were sought and any assumptions and simplifications made.	4 & 5
Critical appraisal of individual sources of evidence	12	If done, provide a rationale for conducting a critical appraisal of included sources of evidence; describe the methods used and how this information was used in any data synthesis (if appropriate).	N/A
Synthesis of results	13	Describe the methods of handling and summarizing the data that were charted.	4 & 5

		Result	
Selection of sources of evidence	14	Give numbers of sources of evidence screened, assessed for eligibility, and included in the review, with reasons for exclusions at each stage, ideally using a flow diagram.	5 & 6
Characteristics of sources of evidence	15	For each source of evidence, present characteristics for which data were charted and provide the citations.	20 to 31
Critical appraisal within sources of evidence	16	If done, present data on critical appraisal of included sources of evidence (see item 12).	N/A
Results of individual sources of evidence	17	For each included source of evidence, present the relevant data that were charted that relate to the review questions and objectives.	20 to 31
Synthesis of results	18	Summarize and/or present the charting results as they relate to the review questions and objectives.	7 to 11
		Discussion	
Summary of evidence	19	Summarize the main results (including an overview of concepts, themes, and types of evidence available), link to the review questions and objectives, and consider the relevance to key groups.	12 to 13
Limitations	20	Discuss the limitations of the scoping review process.	13
Conclusions	21	Provide a general interpretation of the results with respect to the review questions and objectives, as well as potential implications and/or next steps.	13
		Funding	
Funding	22	Describe sources of funding for the included sources of evidence, as well as sources of funding for the scoping review. Describe the role of the funders of the scoping review.	15

Table S2. Search Strategy of Database (Ovid MEDLINE).

#	Searches
1	Antimicrobial resistan$.mp
2	antimicrobial residue$.mp.
3	antibiotic resistan$ bacteria.mp.
4	antibiotic resistance gene$.mp.
5	antimicrobial resistan$ organism$.mp.
6	antibiotic resistant pathogen$.mp.
7	health risk$.mp.
8	1 or 2 or 3 or 4 or 5 or 6 or 7
9	Agriculture$.mp.
10	Farming.mp.
11	fresh agricultural produce$.mp.
12	fresh agriculture product$.mp.
13	fresh vegetable$.mp.
14	raw vegetable$.mp.
15	leafy green$.mp.
16	salad$.mp.
17	fruit$.mp.
18	retail market$.mp.
19	9 or 10 or 11 or 12 or 13 or 14 or 15 or 16 or 17
20	8 and 18 and 19
21	limit 20 to yr="2001 -Current"

Table S3. Types of produce.

Leafy vegetables	Spinach, Lettuce (lamb, Curly lettuce, Iceberg lettuce, Green lettuce, Red lettuce, and Romaine lettuce, Batavia green, Batavia red, Butterhead lettuce, Lollo rosso, Salanova, Lettuce hearts, Lettuce heads, Lettuce little gem), Bok choy, Pak choi, Gai choi, Celery, Chinese nappa, Cilantro, Coriander leaf, herbs, Lollobionda, Arugula, Cabbage, Chinese cabbage, White & red cabbage, Chards, Rucola, Celery, Mint, Parsley, Asiatic pennywort, Water dropwort, Chinese Chives, Chicory, Kyona, Leaf beet, Red chicory, Leaf rape, Curly endive, Water cress, Salad cress, Rocket, Endive, Frisee, Amaranth green, Amaranth red, Water spinach, Laksa leaves, Indian pennywort, Wild parsley, Sweet basil, Japanese parsley, Oakleaf
Non-leafy vegetables	Cucumber, Tomato, Broccoli, Mushrooms, Champignon, Pepper/ Chili, Hot peppers, Long bean, Winged bean, Green beans, Zucchini, Cereals. Leek, Sweet pepper, Green pepper, Artichoke, Lemon
Root vegetables	Bulb onion, Green onions, Spring onion, Welsh onions, Carrot, Chayote, Ginger root, Radish, Turnip, Beet, Garlic, Ginseng
Sprouts	Bean sprouts, Alfalfa seed sprouts, Lentil sprouts, Mung sprouts, Radish seed sprouts, Broccoli sprout, Radish sprout, Rape sprout, Red cabbage sprout, Red kohlrabi sprout, Red radish sprout, Cress, Fenugreek, Lentils, Soybean, Chickpeas sprout
Fruits	Apples, Avocado, Banana, Berries, Cantaloupe (Melon), Mangoes, Kiwi, Watermelon, Peach, Nectarine, Grape, Pears, Strawberries, Oranges, Tangerines, Pomegranates, Cherries, Date, Green table olives, Black table olives,
Other	Fennel, Paprika, Nopalitos (a cactus), Wallflower,
Ready to eat (RTE) mixed salad	Salads in combination of : (lettuce, tomato, onion, cucumber and carrot), (arugula, Batavia green, Batavia red, beet, bok choy, cabbage, carrot, cherry tomato, chicory, cucumber, kyona, leaf beet, lettuce, oak leaf, paprika, perilla, red beet, red bok choy, red cabbage, red chicory, red lettuce and romaine), (cabbage, red cabbage and lettuce), (Batavia green lettuce, arugula, carrot, chicory, frisee salad, spring onion, Batavia red lettuce, and red chicory), (lettuce, beet and purple cabbage), (carrot, purple cabbage and lettuce), (purple cabbage, white cabbage, lettuce and beets)

Table S4. Presence and abundance of ARB and ARGs in fresh produce sold in retail markets.

Data Source (Author/Year)	Items of fresh produce (n)	Key Findings (Presence of resistance bacteria/strains/genes)	Pathogens resistant to antibiotics (test method)	Country
Mustapha Goni Abatcha et al (2018) [9]	Leafy vegetables (405)	• 21.5% of the leafy vegetables were detected with *Salmonella* spp.<br>• Coriander had the highest prevalence (52.0%) of *Salmonella*, followed by lettuce (32.0%), water spinach (31.0%).<br>• 27 *Salmonella* serovars were isolated. *S. weltevreden* was the dominant serovar, followed by S. *corvallis, S. brancaster, S. paratyphi B* and *S. hvittingfoss*.	• 21.8% isolates were resistant to 3 antibiotics.<br>• STR 32 (36.8) had the highest resistance.<br>(disk diffusion)	Malaysia
Sunzid Ahmed et al (2019)[47]	Leafy and non-leafy vegetables (48)	• *E. coli* count in lettuce, tomato and cucumber ranging from 1.69 to 3.75, 1.69 to 1.78 and 0.67 to 3.81 log CFU/g accordingly. 100% coriander leaves contaminated with *E. coli*.<br>• *Staphylococcus* spp. count in lettuce, tomato, cucumber and coriander leaves ranging from 2.30 to 4.52, 2.5 to 3.51, 2.00 to 5.43 and 4.15 to 4.85 log CFU/g accordingly.	• *E. coli* isolated from cucumber and coriander were resistant to AMX, RIF, ERY, Novobiocin, Bacitracin.<br>(disk diffusion)	Bangladesh
Gregor Fiedler et al (2019) [37]	Leafy and non-leafy vegetables (137)	• One hundred and forty-seven *B. cereus*sensulato(s.l) strains were isolated from fresh vegetable samples.<br>• *Bacillus weihenstephanensis, Bacillus cereus, Bacillus thuringiensis, Bacillus toyonensis* species were found from the *B. cereus* s.l. strains.	• Strains showed resistance against the β-lactam antibiotics such as PEN G and CTX (100%), as well as AMC and AMP (99.3%).<br>(disk diffusion)	Germany
Carlos A. Go´mez-Aldapa et al (2016) [23]	Non-leafy vegetables (200)	• Generic *E. coli* were identified in 80% of the 100 whole nopalitos samples and 74% of the 100 cut nopalitos samples.<br>• Diarrheagenic *Escherichia coli* pathotypes (DEPs) were identified in 10% of both types of nopalitos samples.<br>• The identified DEPs included Shiga toxin–producing *E. coli* (STEC), enteropathogenic *E. coli* (EPEC), and enterotoxigenic *E. coli* (ETEC). Respectively, 320 and 280 *E. coli* strains were isolated from whole nopalito samples and cut nopalito samples.	• Among the 20 positive samples of raw and cut Nopalitos, all isolated strains exhibited resistance to at least six antibiotics AMC, AMK, CST, ERY, GEN, KAN. (disk diffusion)	Mexico

Janalíková M et al. (2018) [10]	Leafy, non-leafy, root vegetables and sprouts (216)	• *E. coli* found in 6.9% vegetable samples (Mung sprouts, carrot, zucchini, tomato, baby carrot, raddish, spring onion, lettuce little gem and parsley)<br>• Fifteen strains isolated from raw vegetables.<br>• Antibiotic resistance genes were detected from 13 *E. coli* isolates in this study; they were *qac, sul1, tetA, int, sul1, sul3, mer* and *tetB* (PCR).	• All 15 strains (100.0%) isolated from raw vegetables were resistant to at least one of the twelve tested antibiotics, four of these strains were multi-resistant (3 or more antibiotics), i.e., mung sprouts, raddish and spring onion (disk diffusion)	Czech Republic
Ana Gonzalez et al (2017) [49]	Leafy vegetables (100)	• The highest detection levels of *Arcobacter* spp.were obtained in spinaches 42.90% (9 out of 21). While cabbages showed 25% (1 out of 4), lettuces 14.63 (6 out of 41), and chards 11.76% (4 out of 34); after 48 hours of enrichment in PCR.<br>• 17 out of the 25 isolates were identified as *A. butzleri* and 8 as *A. cryaerophilus.*<br>• In both *A. butzleri* resistant isolates, the sequencing of QRDR fragment revealed the presence of a mutation in position 254 of *gyrA* gene (C-T transition), which was absent in the susceptible isolates and the reference strain *A. butzleri* DSM 8739.	• Only two *A. butzleri* isolates showed resistance to LVX and CIP.<br>(Polymerase chain reaction-restriction fragment length polymorphism)	Spain
Kuan CH et al. (2017) [29]	Leafy, non-leafy and root vegetables (301)	• 58 *Listeria monocytogenes* and 12 *Salmonella enteritidis* were isolated from the vegetable samples<br>• *L. monocytogenes* found from the following number of isolates: carrot (7/58), calamondin (2/58), cucumber (2/58), winged bean (16/58), white raddish (16/58) and cabbage (15/58).<br>• *S. enteritidis* found in carrot (9/12).	• All 58 *Listeria monocytogenes* isolates were found resistant to PEN G. Also, high resistance patterns were observed for MEM (n = 41) and RIF (n = 24).<br>• All the *S. enteritidis* isolates demonstrated resistance to at least four antibiotics (Antibiogram results)<br>• *S. Enteritidis* isolates demonstrated resistance to at least four antibiotics. AMP, AMX, and TMP failed to inhibit all the *S. Enteritidis* strains.<br>• *Salmonella Enteritidis* isolates also displayed high resistance to NA (n=9), SXT(n=9), and CHL (n=8).<br>(disk diffusion)	Malaysia
Siqin Liu et al (2017) [12]	Leafy, non-leafy, root vegetables,	• Leafy vegetables had the highest prevalence of *Enterobacteriaceae* (19.2%), followed	• Antibiotic resistance among the Enterobacteriaceae isolates from the vegetable samples	Tennessee, United States

	sprouts and fruits (360)	by root vegetables (6.4%), tomatoes (5.6%), fruits (3.9%), green peppers (3.3%), and then mushrooms (3.1%).<br>• Broccoli and bean sprouts had the lowest prevalence of *Enterobacteriaceae* (0.8%).<br>• *Shigella* was detected in mushrooms, red lettuce, cilantro, spinach, bokchoy, chayote, squash and bananas.<br>• *Salmonella* at low levels was detected in iceberg lettuce.<br>• None of the sprouts tested were found to be contaminated with *Salmonella, Shigella, or E. coli* O157:H7.	were highest to VAN (99.05%), followed by ERY (91.4%) and AMP (57.6%). None of the isolates were resistant to CIP, AMK, or GEN.<br>(disk diffusion)	
Vera Manageiro et. al. (2020) [44]	Leafy vegetable (1)	• Identified the presence of the plasmid-mediated colistin resistance (PMCR)-encoding gene *mcr-1* in an *Escherichia coli* isolate, INSali25, recovered from a lettuce sample (Whole genome sequencing and Plasmid sequencing)<br>• The presence of additional genes conferring resistance to β-lactams (*bla* $_{TEM-1}$), aminoglycosides (*aadA1, aph(4)-Ia, aph(6)-Id, aac(3)-Iv*), macrolides (*mdf(A)*-type), phenicol (*floR*-type), tetracycline (*tetA*), and sulphonamides (*sul2*) (Silico analysis)<br>• INSali25 isolate showed the *fimH54* and *fumC27* alleles.	• A non-wild-type phenotype to colistin (MIC 16 mg/L) was revealed from the *E. coli* isolate of lettuce sample<br>• The isolate was also resistant to other antibiotic classes, such as PEN, quinolones, AMG, and phenicols, consistent with a multidrug-resistant phenotype.<br>(Microdilution method)	Portugal
Ferielle Mesbah Zekar et al (2019) [25]	Leafy, non-leafy, root vegetables, sprouts and fruits (310)	• Thirteen *Klebsiella pneumoniae* strains resistant to third-generation cephalosporin (3GC) were recovered from mint, tomato, parsley, lettuce, beet, peach, carrot, and celery.<br>• *bla* $_{CTX-M-15}$ in 11 extended-spectrum-beta-lactamases (ESBL)-*K. pneumoniae* and *bla* $_{DHA-1}$ in 2 AmpC-*K. pneumoniae* (Whole genome sequencing)	• *K. pneumoniae* isolates were resistant to some types of penicillin, some first and second generation cephalosporins, cefamandole, some 3GCs (CTX, CAZ and CRO).<br>• 85% of them were resistant to fourth generation cephalosporins cefepime (FEP).<br>• 69% were resistant to AMC and 92% to aztreonam (ATM)<br>• Only 15% were resistant to cefoxitin (FOX).<br>(disk diffusion)	Algeria
J. M. Miranda et al (2009) [32]	Leafy and non-leafy vegetables (78)	• *Salmonella* was detected in 21.8% of the vegetable samples.	• 47.1% of *Salmonella* isolates from vegetables exhibited multidrug resistance.	Mexico

		• The highest prevalence was found for lettuce (33.3%) and carrots (27.3%).	• Three isolates showed maximum resistant to six antibiotics (AMP, CHT, CHL, TET, NA, STR). (Broth microdilution)	
Najwa, M.S. et al (2015) [31]	Leafy and non-leafy vegetables (96)	• The overall prevalence of *Salmonella* spp. was 97.9%. 100% prevalence found in water dropwort, long bean and winged bean. • The overall prevalence of *Salmonella enteritidis* was 54.2%, highest (66.7%) found in long bean. • The overall prevalence of *Salmonella typhimurium* was 82.3%, highest (87.5%) found in water dropwort.	• *Salmonella enteritidis* and *Salmonella typhimurium* isolates were found to exhibit high resistance against AMP (100%), ERY (100%), AMC (81.3%), CHT (75%), STR (50%) and CIP (50%). (disk diffusion) • All *Salmonella* isolates showed multi drug resistant profile	Malaysia
Niyomdecha N 2016 [30]	Leafy vegetable (40)	• 20 % of lettuce had *Salmonella* spp. • *S. panama, S. schwarzengrund, S. rissen* serotypes were found from the lettuce sample.	• *Salmonella* spp. were found resistant to AMP, CHL, SXT, TET, NA (disk diffusion)	Thailand
Reddy S.P 2016 [28]	Leafy and non-leafy vegetables, sprouts (27556 and 111598)	• *Salmonella* prevalence in Cantaloupe 0.03%, Celery 0.02%, Lettuce 0.03%, Tomato 0.01%. (VIDAS assay; data collected from 2001 to 2003) • *Salmonella* prevalence in cilantro were found 0.34%, parsley and spinach 0.29%, hot peppers 0.26%, sprouts 0.25%, Cantaloupe 0.09%, Green onions 0.08%, Lettuce 0.05%, Sprouts 0.25% and Tomatoes 0.02%. (PCR; data collected from 2004 to 2012) • Antimicrobial resistance *Salmonella* serotype found from produce: Oranienburg, Montevideo, Agona, Havana, Thompson, Poona, Kentucky, Tucson, Veneziana serotypes were found from the sample.	• 6.5% (10 of 152) of *Salmonella* isolates from produce showed resistance to antimicrobial compounds. • Antimicrobial resistance *Salmonella* serotypes resistant to CHL, NA, TET, SMX, Trimethoprim (TMP), Cephalothin (CEF), Kanamycin (KAN), STR, AMX, AMP, FOX, Sulfisoxazole (SMX). (National Antimicrobial Resistance Monitoring System gram- negative panel and the Sensititre system.)	USA
Richter L et al 2019 [38]	Leafy and non-leafy vegetables (545)	• Presumptive ESBL producers, predominantly *E. coli, K. pneumoniae, E. cloacae, and E. asburiae* were detected in 17.4% of the vegetable samples analysed.	• 77 selected ESBL-producing *Enterobacteriaceae* isolates were MDR, resistance to AMG (94.8%), CHL (85.7%), and TET (53.2%) antibiotic classes was most prevalent. (disk diffusion)	South Africa
Saksena R 2019 [26]	Leafy, non-leafy, root vegetables and fruits (150)	• *Acinetobacter* spp. was present in onion (18%), cucumber (19%),	• *E. coli, Klebsiella* spp., and *Enterobacter* spp. was resistance	India

		tomato (16%), and chili pepper (18%).<br>• *Klebsiella* spp. was present in onion (11%), cucumber (13%), tomato (11%), chilli pepper (10%) and ginger (12%).<br>• *Enterobacter* spp. was present in onion (10%), cucumber (6%), tomato (8%) and chilli pepper (5%).<br>• The presence of *E. coli* contamination in cabbage was (40%) highest followed by ginger (36%), carrots (27%) and tomato (18%).	to CTX (third-generation cephalosporin [3GC]).<br>• Above 50% of *Acinetobacter* spp. isolates were resistance to ceftazidime (CAZ).<br>(disk diffusion)	
Schwaiger K 2011 [55]	Leafy, non-leafy and root vegetables (1001)	• 722 samples were positive for coliforms (mostly *E. cloacae*; n = 176). *Escherichia coli* were detected in 34, *Pseudomonas* spp. in 439, *Salmonella* spp. in 1, *Enterococcus* spp. in 682, and *Listeria* spp. in 11 samples.<br>• *Pseudomonas* spp. were found by far most frequently in root vegetables such as carrot, celery and raddish (81.6%), 61.2% in leafy vegetables (lettuce and spinach)<br>• *Enterococcus* spp. were found in 77.1% of leafy vegetables and 72.6% of other vegetable e.g., tomato, pepper, zucchini, cucumber.	• High resistance rates were concurrently found for CST in *E. cloacae* from cereals (30%), for cefaclor (CEC) in *E. gergoviae* from salads and cereals (42% and 58%) and for RIF in *E. faecalis* from bulbous vegetables (67%).<br>• *P. aeruginosa* from bulbous vegetables had also significantly higher resistance rates for ceftazidime (CAZ) (9%).<br><br>(microdilution)	Germany
Shah M S et al 2015 [22]	Leafy, non-leafy vegetables and RTE salad (260)	• 34 % vegetable samples were contaminated with *E. coli* strains.<br>• 26.7 % of salad samples were contaminated with *E. coli* strains.<br>• 27.3 %, 31.8% and 18.2 % diarrheagenic *E. coli* strains (DEPs) isolated from cucumber, lettuce and spinach samples respectively.<br>• Enterotoxigenic *E. coli* (ETEC), Enteropathogenic *E. coli* (EPEC), Shiga toxin-producing *E. coli* (STEC) strains were isolated from the positive samples using multiplex (PCR)	• Among the 50 DEPs strains tested, 92 % showed resistance against TET, 87% to AMP, 84% to CTX, 82% to CIP, 79% to CHL, 71% to AZT, 68% to FOX,60% to GEN,58% to TAZ and 10.5 % to IMP.<br>(disk diffusion)	Pakistan
Skočková A et al 2013 [42]	Leafy, non-leafy, root vegetables and sprouts (89)	• *E. coli* detected in 24.7% of the vegetable samples. 22 positive isolates were found. *E. coli* detected the highest in sprouted seed and lettuce sample.<br>Resistant gene isolated from sample: *bla* $_{TEM}$, *tet A*, *tet B*. The *bla* $_{TEM}$ gene was detected in two isolates, the *tet B* gene in three and *tet A* in one. *tet A and tet B conferred resistance to TET,*	• *E. coli* isolates were resistant to AMP, TET, TMP, AMX/ CLA, SMX/TT, SPT and NA.<br>(disk diffusion, E-Test)	Czech Republic

		and bla$_{TEM}$ conferred resistance to AMP and β-lactam antibiotics<br>• isolate detected using PCR.		
Usui M et al 2019 [8]	Leafy, non-leafy, root vegetables and sprouts (130)	• The predominant ESBL producing bacteria *Pseudomonas* spp., isolated from 10 (7.7%) of the vegetable samples followed by *Acinetobacter* spp. (2%), *Rahnella* spp. (1.6%), *Enterobacter* spp. (0.6%) and *Stenotrophomonas* spp. (0.6%).<br>• Following species of ESBL producing bacteria were found from Cabbage: *Pseudomonas hunanensis*, Bean Sprout: *P. putida*, Chinese chive: *P. parafulva*, Broccoli & cabbage: *P. hunanensis*, Onion: *P. beteli*, Cucumber: *P. mosselii*, Ginseng: *P. paralactis, P. arsenicoxydans*.<br>• The ESBL gene detected: *bla* $_{TEM-116,}$ *bla* $_{SHV-12}$ gene were present in the ESBL-producing candidates detected using PCR test	• ESBL producing *Pseudomonas* spp. were resistant to CTX, CHL, CIP, GEN (microdilution)	Japan
Jayde L. Wood et al 2015 [45]	Leafy Vegetable (68)	• *E. coli* detected from 14.7% of the lettuce samples.	• *E. coli* found resistant to AMK, TMP, SXT, NA, AMP, KAN (disk diffusion)	Canada
Fan Yang et al 2019 [43]	Fruits (133)	• 1.5% mcr-1 gene producing bacteria detected from two fruit sample (*E. coli* detected from an apple and *K. pneumoniae* detected from an orange sample)<br>• *E. coli* gene: *aadA2, aadA1, mcr-1, floR, cmlA1, sul2, sul3, tetA, tetM, dfrA12, mdfA*<br>• *K. pneumoniae* gene: *bla* $_{SHV-110}$, *mcr-1, qnrS1, oqxA, oqxB, fosA6, sul1, tetA, dfrA1.*<br>(The analysis was done using Pulsed field gel electrophoresis analysis of S1 nuclease-digested DNA and Southern blotting)	• *E. coli* was resistant to CST and PB; and *K. pneumoniae* was resistant to CST, PB and AMP. (microdilution method).	China
Yingjiao Li et al 2020 [48]	Leafy, non-leafy, root vegetables and sprouts (129)	• The detection rates of *E. coli* were 11.6% in vegetable.<br>• Resistant gene found from vegetable sample *strA, strB, sul2, bla* $_{OXA}$, *tetA, bla* $_{TEM}$, *aadAla, floR, tetB* detected using PCR.	• *E. coli* resistant to TET, AMP, TMP/SXT, AMX, NA, CTX, GEN, CIP, AMK, STR, PIP, CAZ, NA. (disk diffusion)	China

Hong-Seok Kim et al (2015)[40]	Leafy, non-leafy vegetable, roots and sprouts (189)	• The prevalence of ESBL-producing *Escherichia coli* and *Klebsiella pneumoniae* was 10.1% of the total vegetable samples (19 out of the 189 RTE vegetable samples). Of these, 94.7% were from the sprout samples. • Three (15.8%) of the isolates were ESBL-producing *E. coli,* which was all from sprouts, and 16 (84.2%) were *K. pneumonia* with one from mixed vegetable and the other 15 from sprouts.	• All 19 isolates (100.0%) were resistant to CTX, 3 (15.8%) to CAZ, and 2 (10.5%) to FEP. Resistance to ATM was observed in 6 (31.6%) isolates. • All isolates were resistant to AMP and CFZ. • Many of the ESBL producers were also resistant to non-β-lactam antibiotics, including GEN (73.7%), SXT (63.2%), and CIP (26.3%). (microdilution)	Korea
KashinaAllydice-Francis and Paul D. Brown (2012)[33]	Leafy, non-leafy, root vegetables (95)	• 93.3% Lettuce (14/15) and 80% carrots (12/15) were the most frequently contaminated vegetables by *Pseudomonas aeruginosa*, while only 33.3% tomatoes (5/15) were contaminated.	• All isolates were resistant to AMP, and 84% and 83% were resistant to CHL and SXT respectively. • Imipenem (100%), GEN (97%), CIP (93%), and ceftazidime (79%) were the most inhibitory anti- biotics found in this study based on susceptibility results (disk diffusion)	Jamaica
Bao Tao Liu et al 2019 [46]	Leafy, non-leafy, root vegetables (528)	• *mcr-1* gene of *E. coli* detected from 19 (3.6%) fresh vegetable samples. • The detection rate of *mcr-1* of *E. coli* was found in carrot (14.3%), pak choi (13.3%), green pepper (7.7%), leaf lettuce (5.6%), leaf rape (4.9%), romaine lettuce (4.3%), tomato (3.5%), spinach (3.2%), cucumber (3.1%), and curly endive (2.4%). One *mcr-1*-positive *E. cloacae* isolate from green pepper. (S1-pulsed-field gel electrophoresis (PFGE) and Southern Hybridization)	• mcr-1 gene of *E. coli* resistant to CST, AMP, GEN, NA, TET, CIP, CTX, KAN, LVX, DOX, FOS (microdilution)	China
Bezanson G.S et al 2008 [13]	Leafy vegetable and sprouts (205)	• 84.4%, 43% and 40% oxidase-positive, gram-negative bacteria (*Sphingobacterium multivorum, Pseudomonas fluorescens, P. putida, Acinetobacter baumannii*) found from savoy spinach, Romaine lettuce and alfalfa sprout respectively.	• Oxidase positive, gram-negative bacteria resistant to AMP, CEF, CHL, GEN (disk diffusion)	Canada
Christin Freitag et al 2018 [11]	Leafy vegetable and sprouts (245)	• The prevalence of ESBL producing *E. coli* was 2.45% in all vegetable samples. The prevalence	• ESBL producing *E. coli* carried resistant genes which mediated resistance to	Germany

		of ESBL producing *E. coli* was 9.30% of the sprout's samples.<br>• Resistant gene of ESBL producing *E. coli, floR, fosA3, aac (3)-Iva, aph(3')-IIa, aac(6')-Ib3, strA, strB, sul1, sul2, tetA, dfrA14, qnrS1, bla* CTX-M-15,65,125,14,2, *bla* TEM-1 *dfrA1, 14, 17* were detected using PCR.	CHL/FOS, GEN, KAN, STR, SUL, TET, TMP (microdilution and disk diffusion)	
Eva raphael et al 2011 [53]	Leafy vegetable (25)	• Prevalence of bacteria found in spinach sample: *Erwinia persicina* 13.6%, *Pantoea agglomerans* (20.8%), *Pseudomonas putida* 22.4%, *Rahnellaa quatilis* 8% and *Serratia fonticola* 8%.	• *Pseudomonas* spp. resistant to AMP, PEN, SAM, TIM.<br>• *Pantoea agglomerans* resistant to AMP, PEN.<br>• *Rahnella aquatilis* resistant to PEN, AMP, PIP.<br>• *Serratia fonticola* resistant to Cefazolin (CFZ), CEF, Cefuroxime (CXM), Aztreonam (ATM), Cefotetan (CTT), CTX, Ceftazidime (CAZ).<br>• *Erwinia persicina* resistant to PEN, AMP and PIP (microdilution)	USA
Falomir M P et al 2013 [52]	Leafy, non-leafy, root vegetable (160)	• 37% *Enterobacter* species and 18% *Klebsiella* species detected from the total sample	• *Enterobacter* species and *Klebsiella* species resistant to AMC, nitrofurantoin (NIT), TET, STR (disk diffusion)	Spain
Hamilton-Miller et al 2001 [35]	Leafy, non-leafy, root vegetables (isolates from carrot 33; Isolates from salad vegetable 40)	• Bacteria detected from carrot: 36% *P. agglomerans*, 30% *P. fluorescens* and 27% *Rahnella aquatilis*.<br>• Bacteria detected from vegetable sample: 33% *P. agglomerans*, 48% *P. fluorescens.*	• *P. agglomerans, P. fluorescens* and *Rahnella aquatilis* resistant to CTX, TMP, AMP, CXM, CEC, CAZ, AMC. (disk diffusion)	UK
Abriovel H et al 2008 [41]	Leafy, non-leafy, root vegetables and sprouts (102)	• *E. faecalis, E. faecium, E. casseliflavus, E. mundtii* bacteria detected from fruit and vegetable samples.<br>• *E. faecalis* isolated from the sample of green table olives (cracked type), Celery, Tender onion, Cherries, Packed Mediterranean salad, Packed salad (cabbage, red cabbage and lettuce), Green asparagus, Strawberries, Lettuce, Red cabbage, Green pepper, Radish Tomato, Carrot.<br>• *E. faecium* isolated from the sample of green table olives, Beet Artichoke, Alfalfa sprouts, Broccoli, Date, Endive, Packed spring salad,	• *E. faecalis* resistant to ERY, TET, CHL, CIP, LVX, GEN, STR, AMP, PEN<br>• *E. faecium* resistant to quinupristin/dalfopristin, AMP, PEN, CIP, LVX, GEN, CHL, RIF. (ATB ENTEROC 5 strips)	Spain

		Soybean sprouts, Strawberries, tomato, celery		
Juan Luo et al 2017 [24]	Leafy, non-leafy, root vegetables and sprouts (916)	• A total of 244 ESBL-producing *Enterobacteriaceae* isolates (175 *Klebsiella pneumoniae*, 23 *E. coli*, and 46 others) were recovered from 216 samples. • Our results showed that six of the 244 (2.5%) ESBL-producing isolates (four *E. coli* and two *Raoultella ornithinolytica*) were positive for mcr-1. • The prevalence of *mcr-1* in the 23 ESBL-producing *E. coli* isolates was 17.4%. • fosA3, *bla* $_{CTX-M-14}$, *floR*, *oqxAB* resistant gene detected from *E. coli, Raoultella ornithinolytica* bacteria using PCR	• *E. coli* and *Raoultella ornithinolytica* resistant to CST, florfenicol (FFC), CTX, FIF. (microdilution)	China
Ahmed Kabir et al 2015 [50]	Non-leafy, root vegetable (14)	• *Staphlococcus aureus* detected from cabbage in $8x10^2$ CFU/g. • *Pseudomonous* spp. detected from tomato, radish, turnip in 5.4×10^3, 8.0×10^2, $7.2x10^2$cfu/g respectively. • *Listeria* spp. found in cabbage in $6.0x10^2$ CFU/g.	• *S. aureus* resistant to ATM, AMP, Ceftriaxone (CRO), SXT, oxacillin (OXA) • *Pseudomonas* spp. resistant to CRO, CXM, AMP, SXT (Sulfamethoxazol). • *Listeria* spp. resistant to Imipenem (IMP), AMP, GEN, CXM, CRO. (disk diffusion)	Bangladesh
Lori L. Mcgowan et al 2006 [36]	Leafy, non-leafy, root vegetables, sprouts and fruits (240)	• The prevalence of enterococci in tomato and apple were 33.3% and 19.4% accordingly. • The prevalence of enterococci in cucumber, radish and sprouts were 80%, 90.9%, 62.5% respectively. • *E. casseliflavus* was isolated predominantly from apples (66.7%), tomatoes (66.7%), cucumbers (62.5%) and sprouts (60%). • *E. faecalis* detected from apple, tomato, cucumber, sprouts were 16.7%, 22.2%, 25%, 10%.	• Isolates of *E. faecalis, E. faecium* and *E. casseliflavus* resistant to Bacitracin, Flavomycin, Lincomycin. • Isolates of *E. faecalis* and *E. casseliflavus* resistant to CIP. (microdilution)	USA
Ferielle Mesbah Zekar et al (2017) [34]	Leafy, non-leafy, root vegetables and fruits (310)	• 21% (66/310) of the samples found to be positive by 3GC-resistant Gram-negative bacteria. • *Klebsiella pneumoniae* was common in leafy vegetable samples such as celeries, parsleys, mint and lettuce. Also found in tomato. • *E. cloacae* was found in fruit samples such as watermelons,	• All of the isolates were resistant to AMP and TIC. • *Klebsiella pneumoniae* isolates found in carrot were resistant to a maximum of 20 antibiotics and minimum 12 antibiotics from tomato sample. • Again, *E. cloacae* found in carrot were resistant to a	Algeria

		peach, tomato, pepper, nechtarine, pear, grape, chilli, cucumber, apple.<br>• Both *E. cloacae* and *Klebsiella pneumoniae* found in few samples of root vegetables such as carrot and beet.	maximum of 15 antibiotics and minimum 11 antibiotics in apple sample.<br>(disk diffusion)	
Meher Nipa et al (2011) [54]	Leafy, non-leafy, root vegetable (32)	• All the vegetables were highly contaminated with<br>• Coliform and fecal Coliform (> 1100 CFU/100ml).<br>• *Enterobacter* spp. (21.80%) was the most dominant followed by *Pseudomonas* spp. (19.17%), *Vibrio* spp. (16.92%), *Lactobacillus* spp. (15.04%), *Staphylococcus* spp. (10.15%), *Klebsiella* spp. (9.04%), *E. coli* (4.89%), *Citrobacter* spp. (2.26%), *Serratia* spp. (0.37%) and *Salmonella* spp. (0.37%).	• *Pseudomonas* spp., *Enterobacter* spp., resistant to ERY, GEN, AMP, CIP, cephalexin (LEX), CHL, STR.<br>• *E. coli* spp., *Vibrio* spp., *Lactobacillus* spp., *Klebsiella* spp. resistant to ERY, GEN, AMP, cephalexin (LEX), CHL, STR.<br>(disk diffusion)	Bangladesh
Ramona Iseppi et al (2018) [27]	Leafy, root vegetables (160)	• A total of 312 Gram-negative strains were isolated, of which 160 isolates were recovered from fresh vegetables, with a high proportion of *Citrobacter freundii* (26%).<br>• The other strains identified were *E.cloacae* (22%), *Pantoea agglomerans* (16%), *Rahnella aquatilis* (12%), *E. coli* (10%), *Hafnia alvei* (5%), *Enterobacter aerogenes* (5%), and *Pseudomonas fluorescens* (4%).<br>• The remaining 152 isolates were recovered from RTE salads, with a high prevalence of *E. cloacae* (26.3%) and *E. coli* (25%). The other strains identified were *E. aerogenes* (22.3%), *P. fluorescens* (7.9%), *Klebsiella ozaenae* (7.9%), *P. agglomerans* (5.3%), and *C. freundii* (5.3%).<br>• In the bacterial isolates, the resistant genes present in vegetables mainly belonged to the CTX-M family, and more specifically, CTX-M-15 was found both in fresh vegetables and in RTE salads.	• *Rahnella aquatilis P. agglomerans* and *E. cloacae* resistant to AMP, AMC, CTX.<br>• *Escherichia coli* resistant to AMP, AMC, CTX, CAZ.<br>• *C. freundii* resistant to AMP, CTX.<br>• *K. ozaenae* resistant to AMP, FOX, CTX.<br>(microdilution)	Italy
Angela H.A.M. van Hoek et al (2015) [6]	Leafy, root vegetables (1216)	• From 63 (5.2%) of the 1216 samples analysed 3rd generation cephalosporins (3GC) resistant *Enterobacteriaceae* could be isolated.<br>• *Enterobacter* spp. were detected in 33.3% (21/63) of all positive items,	• 100% (43) of ESBL isolates were resistant to Ampicillin. Among ESBL-producing isolates, AMC resistance was only observed among *Enterobacter* spp. (87.5%).	Netherlands

		and highest found in spring onion 60% (6/10) sample.<br>• *Serratia* spp. were detected in 44.4% (28/63) of all positive items, and highest found in Butterhead lettuce 77.8% (7/9) sample<br>• *E. coli* detected 11.1% in Blanched celery (1/9).	• 95.2% (20 out of 21) of AmpC isolates were resistant to AMC and CTX.<br>(disk diffusion)	
Vanessa de Vasconcelos Byrne et al (2016) [39]	Leafy, non-leafy and root vegetables (99)	• *L. monocytogenes* was isolated from four out of 99 (4.04%) samples, of which one came from raw vegetables (2.22%) and three (5.56%) came from ready-to-eat vegetables (salads), Lettuce, beets and purple cabbage (1 sample), Lettuce, beets, purple cabbage and white cabbage (2 samples).	Two *L. monocytogenes* isolates from ready-to-eat vegetables exhibited resistance to PEN G and, TET.<br>(disk diffusion)	Brazil

Note: multidrug resistance meant resistance to three or more antibiotics. Amoxicillin (AMX), Ampicillin (AMP), ampicillin-sulbactam (SAM), Aminoglycoside (AMG), Amikacin (AMK), amoxicillin-clavulanic acid, Co-amoxiclav (AMC), Aztreonam (ATM), Clavulanic acid (CLA), ceftriaxone (CRO), cephalotin (CHT) Cefoxitin (FOX), Cefuroxime (CXM), Cefotaxime (CTX), Chloramphenicol (CHL), ceftazidime (CAZ), Colistin (CST), Cefaclor (CEC), Ciprofloxacin (CIP), Cephalothin (CEF), Cefoperazone (CFP), cefazolin (CFZ), Cefotetan (CTT), Cephalexin (LEX), Doxycycline (DOX), Fosfomycin (FOS), Erythromycin (ERY), Gentamicin (GEN), imipenem (IPM), Kanamycin (KAN), Levofloxacin (LVX), Nalidixic acid (NA), Nitrofurantoin (NIT), Oxacillin (OXA), Polymyxin B (PB), Penicillin (PEN), Piperacillin (PIP), Piperacillin-tazobactam ((TZP), Rifampicin (RIF),Sulfamethoxazole (SMX), Sulfisoxazole (SX), Streptomycin (STR), Sulphonamides (SUL), Trimethoprim-sulfamethoxazole (SXT), Ticarcillin-clavulanic acid (TIM), Trimethoprim (TMP), Tetracycline (TET).
